# A Case Series Characterizing the Spectrum of Colonic Perforation: A Retrospective Study From a Tertiary Care Center in Central India

**DOI:** 10.7759/cureus.92986

**Published:** 2025-09-22

**Authors:** Anjani Goli, Krishnanand Anand, Abhimanyu Yadav, Priya S Kushwah

**Affiliations:** 1 Department of General Surgery, LN Medical College and Research Center, Bhopal, IND

**Keywords:** acute colonic perforation, hartmann procedure, intestinal perforation, perforated colon, primary colonic repair, resection and anastomosis, spontaneous colonic perforation, spontaneous intestinal perforation, surgical management colonic perforation, traumatic perforation

## Abstract

Background

Colonic perforation is a rare but life-threatening surgical emergency with a broad spectrum of etiologies and presentations. This retrospective case series from a tertiary care center in Central India analyzes 15 consecutive cases to describe patient demographics, clinical features, diagnostic findings, perforation sites, etiologies (including iatrogenic and colonoscopy-related), management strategies, and outcomes, with an emphasis on early recognition and tailored surgical intervention.

Methodology

This retrospective, observational study included adults with confirmed colonic perforation treated between January 2022 and March 2025 at a tertiary care hospital in Central India. Data collection encompassed demographics, clinical presentation, duration of symptoms, diagnostic workup (imaging modalities, including X-ray and CT, and colonoscopy when available), perforation characteristics (site, suspected etiology, etc.), intraoperative findings, and definitive surgical management and its outcomes. Descriptive statistics were employed to summarize patterns and outcomes, with particular attention to associations among perforation site, cause, and chosen surgical strategy.

Results

The cohort showed a female predominance of 9/15 (60%), with 60% aged 60 years or older. All patients presented with abdominal pain; tenderness was universal, 15/15 (100%), with abdominal rigidity in 86.6% (13/15) and peritoneal signs in most cases. Pneumoperitoneum was detected on imaging in all patients, 15/15 (100%). The transverse colon was the most frequently involved site, 8/15 (53.3%), followed by the sigmoid, 3/15 (20%), and descending colon, 2/15 (13.3%). Etiologies included iatrogenic, colonoscopy-related perforations, as well as infectious, inflammatory, and spontaneous causes. Imaging consistently demonstrated pneumoperitoneum, with CT and X-ray aiding localization and planning. All patients underwent surgery, with management strategies including Hartmann’s procedure, 6/15 (40%), primary repair, 5/15 (33.3%), and primary repair with diverting stoma, 3/15 (20%), decisions guided by the degree of contamination and patient stability. The outcomes underscore the importance of prompt surgical intervention and individualized decision-making to optimize results in this setting.

Conclusions

Early recognition and meticulous, patient-specific surgical management are essential for favorable outcomes in colonic perforation. The transverse and sigmoid colon emerged as common sites, reflecting anatomical vulnerabilities and procedure-related risks in this cohort. Cross-sectional imaging facilitates rapid localization and planning, enabling appropriate selection among primary repair, diversion, or resection with stoma. This study reinforces the value of a multidisciplinary, tailored approach to reduce morbidity and mortality associated with colonic perforation in resource-limited, high-volume centers, emphasizing the potential for improved survival through timely, evidence-informed clinical decisions.

## Introduction

Colonic perforation can result from a broad spectrum of etiologies, including intrinsic colorectal pathologies, procedural complications, iatrogenic trauma, or infectious etiologies. Disease-related perforations frequently occur in the context of severe inflammatory or neoplastic processes. These include perforation secondary to acute inflammatory conditions, such as appendicitis, diverticulitis, or advanced colorectal carcinoma, where transmural necrosis and erosion of the bowel wall may lead to fulminant perforation [1,2]. In such scenarios, perforation typically involves rupture of inflamed, ischemic, or necrotic tissue, leading to spillage of luminal contents into the peritoneal cavity.

Colonic perforation remains a formidable surgical emergency with significant implications for patient morbidity and mortality, especially in the context of a tertiary care referral center where complex and advanced colorectal cases present frequently [3]. This case series, conducted over more than 36 months at a high-volume tertiary referral institution, aims to provide comprehensive insights into the diverse etiologies, clinical presentations, and surgical management strategies employed for colonic perforation in a specialized healthcare setting.

Additional critical inflammatory conditions, such as toxic megacolon arising in the course of severe ulcerative colitis, can result in colonic wall ischemia and subsequent perforation owing to sustained distention and transmural inflammation [4]. Infectious colitis, particularly *Clostridium difficile* infection, can compromise mucosal integrity, causing necrosis and predisposing the bowel to perforation [5].

A significant proportion of colonic perforations are iatrogenic, occurring during diagnostic or therapeutic colonoscopy. The overall incidence remains low, approximately 0.05% to 0.39%, but the potential consequences are grave [4]. Perforation during colonoscopy requires urgent recognition and typically mandates surgical intervention for definitive repair, especially in cases of significant contamination, delayed diagnosis, or failure of endoscopic closure. The development and increased application of advanced endoscopic techniques, such as endoscopic submucosal dissection, have expanded therapeutic options but concurrently elevated the risk of perforation [4]. These procedures involve intricate dissection and mucosal manipulation that can compromise the integrity of the colonic wall, especially in friable tissues [4].

Given the increasing complexity of colonoscopic procedures, a thorough understanding of the clinical presentation, risk stratification, and immediate management strategies for colonic perforation is essential. Surgical intervention remains the mainstay of treatment in most cases, involving exploratory laparotomy or laparoscopy, extensive peritoneal decontamination, assessment of bowel viability, and tailored resections [3,6]. The decision to perform primary anastomosis versus staged procedures such as resection with colostomy hinges on intraoperative findings, degree of contamination, and patient stability [3].

This study describes 15 cases of colonic perforation, each exhibiting unique clinical features. The heterogeneous presentation underscores the importance of individualized surgical management strategies in achieving optimal patient outcomes in the context of this potentially life-threatening surgical emergency [7]. Through this detailed analysis of 15 cases over more than two years, our goal is to contribute valuable insights to the existing literature and to inform best surgical practices for managing this ominous emergency in complex healthcare settings [8].

## Materials and methods

Study design and inclusion/exclusion criteria

This retrospective, observational study was conducted to evaluate the clinical characteristics, diagnostic approaches, management, and outcomes of colonic perforation cases, each with unique clinical features. The study period extended from January 2022 to March 2025. The study population consisted of 15 patients diagnosed with colonic perforation who were treated at a tertiary care hospital, which included patients diagnosed with colonic perforation based on clinical, radiological, and intraoperative findings. Patients aged >18 years and older were considered for inclusion. Patients were included if complete medical records, including laboratory and imaging data, operative notes, and postoperative follow-up data, were available. All patients aged <18 years, those with multiple perforations at different sites (other than the colon), pregnant women, and those with trauma-induced perforation were excluded from the study.

Data collection

Data were collected from medical records, focusing on demographics (age, gender, residential background, comorbidities), clinical presentation (symptoms, vital signs, symptom duration), diagnostic evaluation (laboratory tests, X-rays, CT scans), management (surgical type, intraoperative findings, postoperative care), and outcomes (recovery, complications, mortality, and follow-up, including colostomy reversal).

Ethical considerations

This study was conducted in accordance with ethical standards and approved by the Institutional Ethics Committee, L.N. Medical College and Research Center and JK Hospital (approval number: 160). To ensure patient confidentiality and comply with ethical standards, all identifying information was anonymized before data analysis. Personal identifiers such as names, exact dates of procedures, and specific location details were removed or coded, maintaining the privacy of the patients while allowing for accurate data collection and analysis. This approach safeguarded patient anonymity throughout the retrospective review of the case series. Informed consent was not required due to the retrospective nature of the study, and consent was taken wherever necessary.

## Results

The analysis of 15 patients with colonic perforation (including patients following colonoscopic perforation) provides detailed insights into the demographics, clinical presentation, imaging findings, perforation sites, and management approaches.

Of the patients studied, 40% (4/15) were male and 60% (9/15) were female, with ages ranging from 28 to 75 years. A significant proportion, 60% (9/15), were 60 years or older, suggesting that advanced age may contribute to the risk of perforation. Younger patients (<60 years) constituted 40% (6/15) of the cases, indicating that while age plays a role, it is not the sole determinant (Table 1).

**Table 1:** Distribution of findings in colonic perforations (n = 15).

Variable	Details	Frequency	Percentage (%)
Gender	Male	6	40%
	Female	9	60%
Age	≥60 years	9	60%
	<60 years	6	40%
Symptoms	Abdominal pain	15	100%
	Vomiting	10	66.60%
	Dyspnea	5	33.30%
	Nausea	9	60%
	Fever	7	46.60%
Signs	Decreased bowel function	11	73.30%
	Tenderness	15	100%
	Rigidity	13	86.60%
	Distention	9	75%
	Bowel sounds absent	12	80%
Time of symptom onset	1–3 days	8	53.30%
	5–9 days	5	33.30%
	>9 days	2	13.30%
Imaging findings	Pneumoperitoneum	15	100%
Colonoscopy	Post-colonoscopy induced	4	26.60%
Iatrogenic		4	26.60%
Site of perforation	Ascending colon	0	0%
	Transverse colon	8	53.30%
	Descending colon	2	13.30%
	Sigmoid colon	3	20%
Management	Laparotomy with Hartmann’s procedure (end colostomy)	6	40%
	Laparotomy with primary closure of perforation	5	33.30%
	Laparotomy with primary closure and ileostomy	3	20%

Clinical presentation

**Figure 1:**
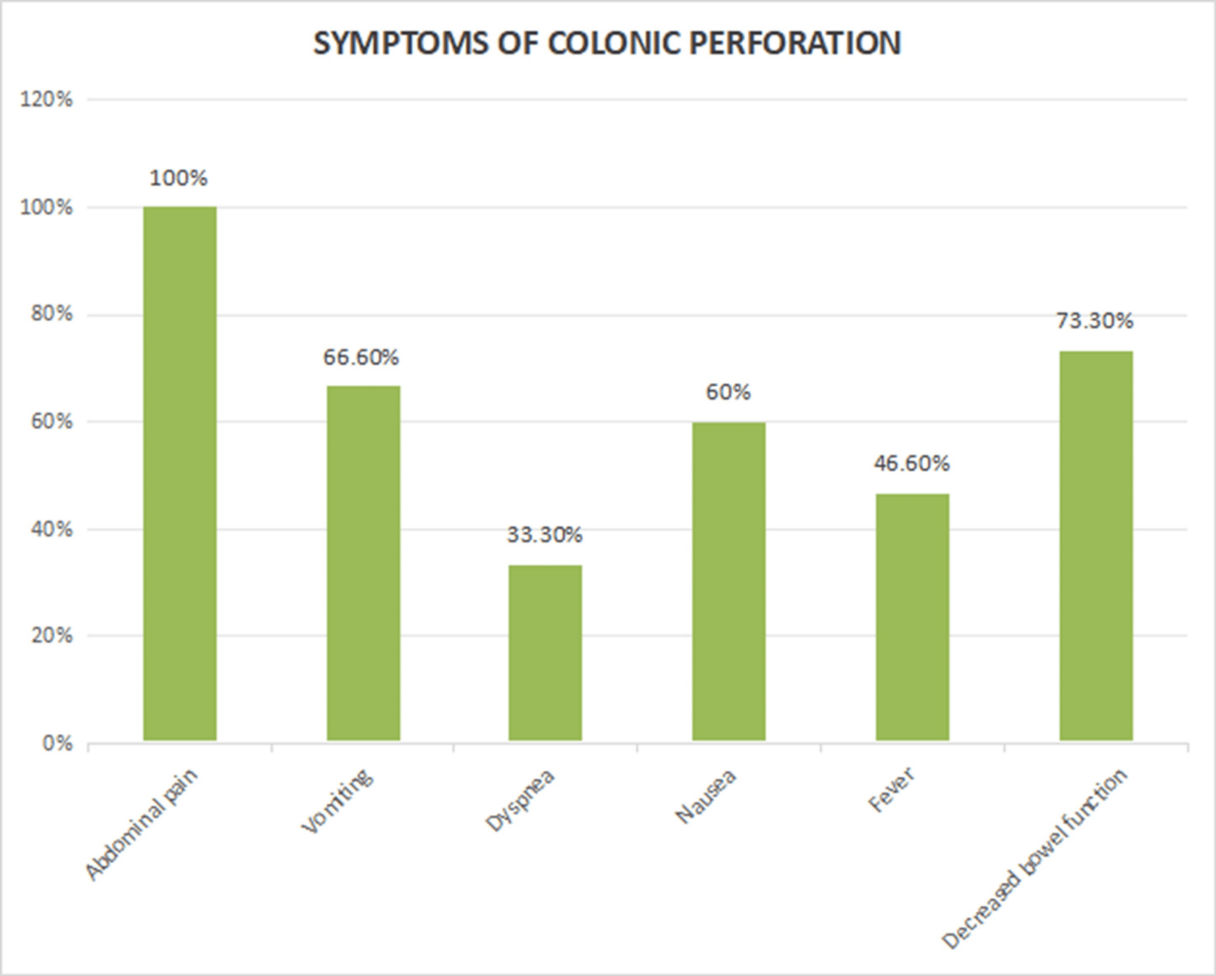
Symptoms of colonic perforation.

**Figure 2:**
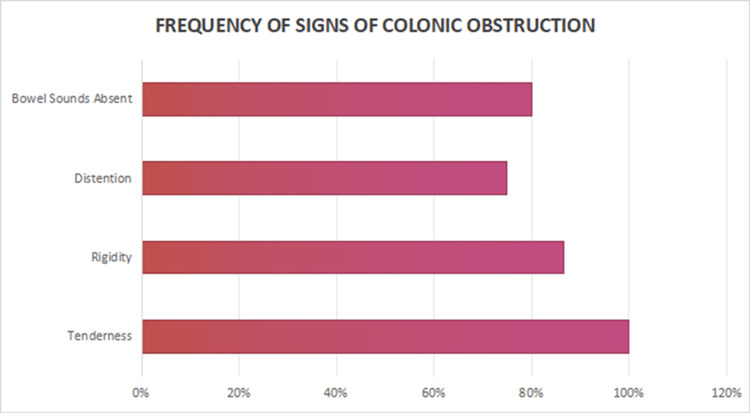
Frequency of signs observed in colonic perforation.

The clinical presentation varied among patients, though abdominal pain and decreased bowel function were overwhelmingly the most common symptoms, present in the majority of the cases, followed by vomiting in 10 (66.6%), nausea in 9 (60%), fever in 7 (46.6%), and dyspnea in 5 (33.3%). On clinical examination, tenderness was present in all cases (100%), rigidity in 13 (86.6%), absent bowel sounds in 12 (80%), abdominal distention in 9 (75%), and decreased bowel function in 11 (73.3%), indicating significant peritoneal irritation and bowel compromise (Figure 1). The time of onset of symptoms following the procedure ranged widely, with 8 (53.3%) patients presenting within one to three days, especially seen in patients who underwent colonoscopy and transverse colonic perforation, 5 (33.3%) between five and nine days, and 2 (13.3%) after nine days (Figure 2). This variation underscores the need for vigilance over an extended period following colonoscopy or in patients with risk factors.

Imaging findings

**Figure 3:**
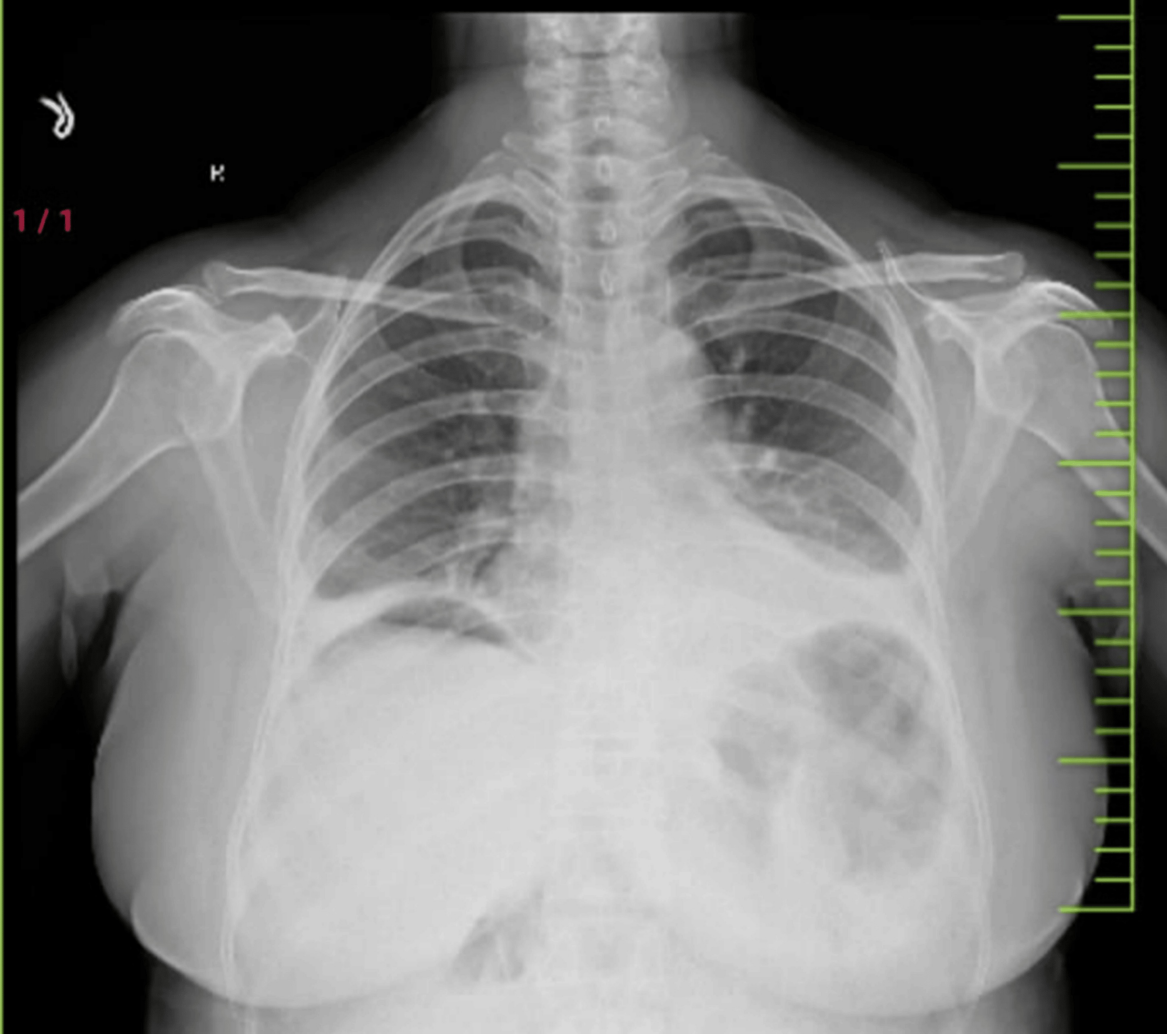
X-ray erect abdomen of a 53-year-old female patient showing gas under the diaphragm on the right side.

Imaging findings were crucial for the diagnosis and characterization of perforations. Pneumoperitoneum was the most frequently observed abnormality, present in 15 (100%) cases (Figure 3). These findings highlight the importance of imaging studies, such as X-ray and CT, in identifying complications and guiding management decisions.

Site of perforation

**Figure 4:**
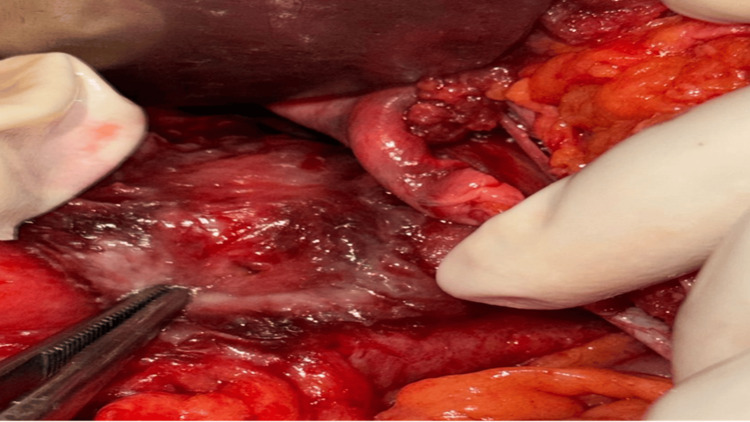
Colonic perforation seen along the retroperitoneal surface of a splenic flexure of the transverse colon measuring approximately 1 cm.

**Figure 5:**
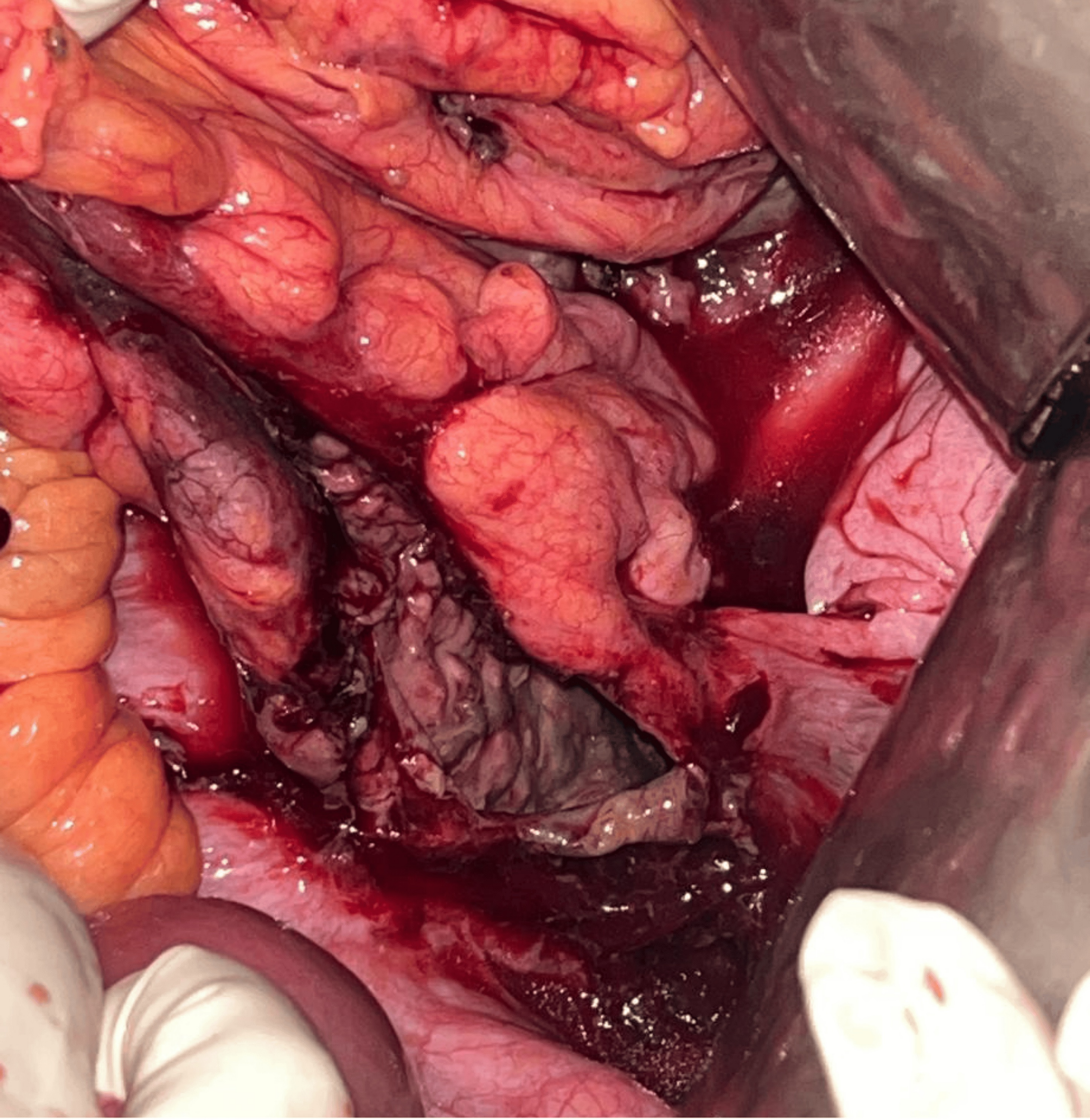
Colonic perforation due to gangrene of the colon involving a transverse colon splenic flexure and the descending colon.

**Figure 6:**
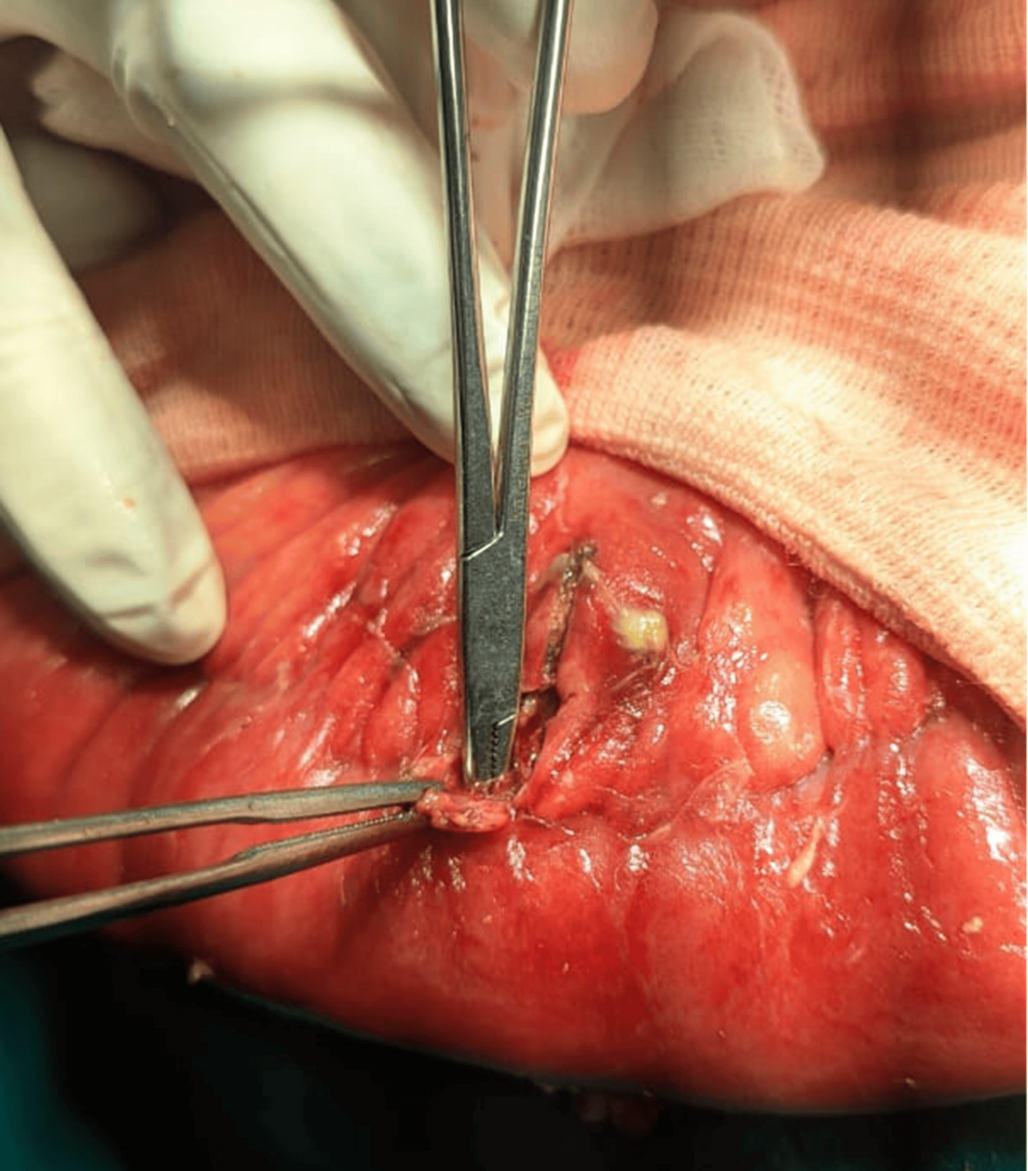
Colonic perforation seen along the mesenteric border of the sigmoid colon.

The site of perforation was most commonly the transverse colon, accounting for 8 (53.30%) cases, followed by the sigmoid colon, which was involved in 3 (20%) patients. This distribution suggests that the transverse colon may be more vulnerable to perforation, possibly due to its anatomical and functional characteristics. Within the transverse colon, the splenic flexure appeared to be more prone to injury, especially in cases of infectious origin (gangrene of the splenic flexure) and iatrogenic (such as percutaneous nephrolithotomy) (Figures 4, 5). The sigmoid colon was more vulnerable to perforation, especially during colonoscopy (Figure 6). Colonoscopy-induced perforations were more commonly caused in the sigmoid colon, followed by the rectum or the descending colon.

Management

Management approaches in this study were individualized based on the severity, location, and underlying cause of the colonic perforation, as well as the patient’s overall clinical condition. All 15 (100%) patients required surgical intervention, reflecting the emergent nature of this condition. Surgical procedures included laparotomy with primary repair of the perforation (33.30%; 5/15), which was performed in select patients where the contamination was limited and bowel viability was preserved. In some of these cases, a protective ileostomy (20%; 3/15) was added to divert fecal flow and reduce anastomotic risk. For patients presenting with more extensive contamination, hemodynamic instability, or poor bowel condition, a laparotomy with colostomy or ileostomy, most commonly Hartmann’s procedure (40%, 6/15), was performed. This involved resection of the perforated segment with closure of the rectal stump and creation of an end colostomy, offering a safer alternative in critically ill patients. The choice of procedure was determined intraoperatively based on the extent of peritoneal contamination, bowel viability, and patient stability.

## Discussion

Colonic perforation is a comparatively uncommon but serious complication that requires prompt diagnosis and tailored management to improve outcomes. In the present study, the analysis of 15 cases provided insights into demographic factors, clinical presentations, imaging findings, perforation sites, and management strategies, which are consistent with existing literature.

The demographic distribution highlighted a predominance of female patients, 9/15 (60%), and an association with advanced age (≥60 years in 60%) [1,2]. This aligns with findings from previous studies indicating that older individuals are at a higher risk of perforation due to age-related changes in colonic structure and comorbidities [3]. However, 6/15 (40%) of the cases involved younger patients, underscoring the multifactorial nature of this complication.

Abdominal pain and vomiting were the most common symptoms, occurring in approximately 90% of patients, while other presentations included fever, dyspnea, and nausea. Tenderness and rigidity were seen in almost all patients preoperatively. Other signs included distention and decreased bowel sounds. The variable time of symptom onset, ranging from one to over nine days, indicates that close monitoring should be maintained for an extended period, especially in high-risk populations, such as postoperative or post-colonoscopic status [4].

Imaging played a critical role in diagnosis, with pneumoperitoneum the most frequent finding, further demonstrating the value of CT scans in detecting complications and guiding clinical decisions. Early imaging following the detection of suspicious symptoms is crucial, as delayed recognition can worsen outcomes [5].

The transverse colon was the most common site of perforation in 8/15 (53.3%) patients, followed by the sigmoid colon in 3/15 (20%). This pattern corroborates findings by Panteris et al. [6], who noted that the sigmoid colon is particularly vulnerable due to shearing forces during scope insertion, the presence of diverticula, and/or polyps. Additionally, the thin muscular layer and larger diameter of the cecum make it susceptible to barotrauma [7]. A history of ulcerative colitis with friable colonic mucosa, as seen in one patient, is another predisposing factor for perforation [8].

This study has several limitations, including a small sample size of only 15 patients and a single-center design, which restricts the generalizability of the findings. The retrospective design is prone to selection and documentation biases. Surgical decisions were made intraoperatively without a standardized protocol and involved multiple surgeons, introducing variability in management. The study also lacked a control group and long-term follow-up data, making it difficult to assess the comparative effectiveness and durability of the different surgical approaches. Additionally, the inclusion of heterogeneous etiologies further limits the applicability of the results to specific patient subgroups.

Management strategies were tailored based on the severity of the condition and underlying pathology. Surgical intervention was required with procedures ranging from primary closure to Hartmann’s procedure, which is consistent with recommendations for patients with minimal symptoms and stable conditions [9,10]. The choice of different surgical management depends on several factors, including the site of injury, bowel preparation quality, and the patient’s clinical stability [11,12]. The primary principle of surgery will be to control the offending pathology, reestablish bowel continuity (only in a stable patient while minimizing any untoward complications) or provide an ostomy (diversion or end-stoma), and mitigate any long-term adverse outcomes [13,14].

## Conclusions

Colonic perforation, although relatively rare, represents a life-threatening emergency that necessitates early diagnosis and prompt, individualized intervention to minimize morbidity and mortality. The findings from this 15-case study reinforce the importance of maintaining a high index of suspicion, especially in elderly and female patients, who may be more vulnerable due to age-related anatomical and physiological changes. Clinical vigilance is particularly critical in postoperative and post-endoscopic patients, where symptoms may present late or atypically. Cross-sectional imaging, especially CT scans, played a pivotal role in early identification of perforation, commonly revealing pneumoperitoneum and guiding operative planning. The transverse and sigmoid colon emerged as the most frequently involved segments, likely due to their anatomical positioning and increased susceptibility during procedures. Early surgical intervention, preferably within the first 24 hours, was associated with significantly better outcomes. Treatment strategies varied based on the site of perforation, degree of peritoneal contamination, and patient stability, including options such as primary repair, resection with diversion, or Hartmann’s procedure. Overall, this study highlights that optimal outcomes depend on early detection, aggressive source control, and a multidisciplinary, patient-tailored surgical approach aimed at preserving bowel function and reducing postoperative complications.

## References

[1] Kidogawa H, Nonomura R, Uehara T, Shinyama S, Okamoto K (2024). Two cases of colonic perforation following upper gastrointestinal barium examinations. Cureus.

[2] Satani T, Watanabe Y, Shimazaki J (2015). [A case of colorectal perforation after barium gastrography]. J Japan Soc Coloproctol.

[3] Matsuo R, Fukuzawa J, Ikeda O, Nakano J, Nakayama K (2015). [A case of rectal perforation after gastrography]. J Japanese Soc Clin Surg.

[4] Okada A, Aotake T, Doi K, Tanaka F, Fujii H, Hirose Y (2015). [A case of descending colon perforation caused by barium after gastric cancer screening]. J Japanese Soc Clin Surg.

[5] Kudo K, Suenaga Y, Kawamoto K, Iwagaki T, Sato H (2015). [A case of sigmoid colon perforation due to upper gastrointestinal radiography cured by conservative therapy]. J Japanese Soc Clin Surg.

[6] Uchida E, Izumi M, Tsuchiya I (2013). [A case of sigmoid colorectal perforation due to barium retention after gastrography]. Prog Dig Endosc.

[7] Hamasaki K, Kawagoe K, Shibuya A, Fukuoka H, Sumida Y, Ishikawa H (2015). [A case of colon perforation after an upper gastrointestinal barium series]. J Japanese Soc Clin Surg.

[8] Sudo N, Kobayashi T, Hirose Y, Katada T, Takizawa K, Wakai T (2015). [A case of survival after cardiopulmonary arrest caused by barium peritonitis due to colorectal perforation following upper gastrointestinal radiography]. J Japanese Abdom Emerg Med.

[9] Kinoshita M, Kataoka M, Tashiro M, Kato K, Kondo K (2015). [A case of stercoral colonic perforation by barium in a young man with no underlying disease]. J Japanese Abdom Emerg Med.

[10] Kashiwabara T, Mizukami Y (2016). [A case of rectal perforation without organic disease after upper gastrointestinal radiography]. J Compr Health Screen.

[11] Pantel H, Reddy VB (2023). Management of colonic emergencies. Surg Clin North Am.

[12] Jaboury IA (2018). Idiopathic colonic perforation in adults. ANZ J Surg.

[13] Zurbuchen EA, Sela N, Maskin A (2021). Transverse colonic perforation in renal transplant recipients during the early postoperative period: a case series. Transplant Proc.

[14] Lüning TH, Keemers-Gels ME, Barendregt WB, Tan AC, Rosman C (2007). Colonoscopic perforations: a review of 30,366 patients. Surg Endosc.

